# Increased expression of the immune modulatory molecule PD-L1 (CD274) in anaplastic meningioma

**DOI:** 10.18632/oncotarget.3082

**Published:** 2014-12-31

**Authors:** Ziming Du, Malak Abedalthagafi, Ayal A. Aizer, Allison R. McHenry, Heather H. Sun, Mark-Anthony Bray, Omar Viramontes, Revaz Machaidze, Priscilla K. Brastianos, David A. Reardon, Ian F. Dunn, Gordon J. Freeman, Keith L. Ligon, Anne E. Carpenter, Brian M. Alexander, Nathalie Y. Agar, Scott J. Rodig, Elizabeth M. Bradshaw, Sandro Santagata

**Affiliations:** ^1^ Department of Pathology, Brigham and Women's Hospital and Harvard Medical School, Boston, Massachusetts, USA; ^2^ Department of Radiation Oncology, Brigham and Women's Hospital and Harvard Medical School, Boston, Massachusetts, USA; ^3^ Department of Neurology, Brigham and Women's Hospital and Harvard Medical School, Boston, Massachusetts, USA; ^4^ Imaging Platform, The Broad Institute of MIT and Harvard, Cambridge, Massachusetts, USA; ^5^ Department of Neurosurgery, Brigham and Women's Hospital and Harvard Medical School, Boston, Massachusetts, USA; ^6^ Department of Medical Oncology, Dana-Farber Cancer Institute and Harvard Medical School, Boston, Massachusetts, USA; ^7^ Department of Neuro-Oncology, Massachusetts General Hospital and Harvard Medical School, Boston, Massachusetts, USA; ^8^ Department of Cancer Biology, Dana-Farber Cancer Institute and Harvard Medical School, Boston, Massachusetts, USA

**Keywords:** meningioma, PD-L1, RNAscope, immunotherapy

## Abstract

There are no effective medical treatments for WHO grade III (anaplastic) meningioma. Patients with this high-grade malignancy have a median survival of less than two years. Therapeutics that modulate the mechanisms that inhibit local immune responses in the tumor microenvironment are showing significant and durable clinical responses in patients with treatment refractory high-grade tumors. We examined the immune infiltrate of 291 meningiomas including WHO grade I-III meningiomas using immunohistochemistry and we examined the expression of PD-L1 mRNA by RNAscope in situ hybridization and PD-L1 protein by immunohistochemistry. In meningioma, the tumor infiltrating lymphocytes are predominantly T cells. In anaplastic meningioma, there is a sharp decrease in the number of T cells, including the numbers of CD4+ and CD8+ T cells and cells expressing PD-1 and there is also an increase in the number of FOXP3 expressing immunoregulatory (Treg) cells. PD-L1 expression is increased in anaplastic meningioma – both mRNA and protein. Using patient derived meningioma cell, we confirm that PD-L1 is expressed in meningioma cells themselves, and not solely in infiltrating immune cells. This work indicates that high-grade meningioma harbor an immunosuppressive tumor microenviroment and that increased Treg cells and elevated PD-L1 may contribute to the aggressive phenotype of these tumors.

## INTRODUCTION

Meningiomas are the most common primary intracranial tumor accounting for over one third of all brain tumors [1]. In the United States, the incidence of pathologically-confirmed meningioma is approximately 7.4 per 100,000 individuals with a prevalence of 97.5 per 100,000 individuals – there are approximately 18,000 new cases diagnosed annually and 170,000 people living with a diagnosis of meningioma [1, 2]. Most meningioma are WHO grade I tumors and can be treated effectively with surgery, however, a subset have more aggressive features. Over 20% of meningioma are WHO grade II (atypical) tumors [3] and approximately 3% are WHO grade III (anaplastic) meningioma [4]. Patients with WHO grade II or III meningiomas are significantly more likely to have a local recurrence after their initial treatment and moreover have a shorter overall survival compared to patients with WHO grade I meningioma [5]. Reported recurrence rates vary widely across published reports but there is a strong association of recurrence with WHO grade: 7-20% for WHO grade I meningioma, 30-40% for atypical meningioma and 50-94% for anaplastic meningioma [3, 6-9]. Notably, the prognosis for anaplastic meningioma is much worse than for atypical meningioma – in one large study atypical meningioma had a 5-year mortality rate of 21% while anaplastic meningioma had a 5-year mortality rate of 68% with a median survival of only 1.5 years [6].

Options for blocking meningioma growth with targeted therapeutics appear possible for only a small subset of patients – for example, selective inhibitors of SMO and AKT may prove to be useful for patients with tumors harboring driver mutations in those oncogenes. Such meningiomas appear to be most frequently WHO grade I tumors that arise in the skull base [10, 11]. Meningiomas also frequently harbor mutations in NF2 [12-16] as well as in TRAF7 and in KLF4 [10, 17]. A subset of meningioma (the angiomatous subtype) harbor chromosomal polysomies [18] and the clear cell subtype can have mutations in SMARCE1 [19]. High-grade meningioma characteristically bear recurrent whole arm chromosomal losses [20-24] and TERT promoter mutations associated with histological progression [25]. Because none of these additional genetic aberrations can currently be targeted with selective therapeutics, strategies using broadly active agents may be needed for effective disease-management in most patients with high-grade meningioma.

Insights into the molecular mechanisms underlying how the immune system responds to tumors has uncovered the important role of immune checkpoint pathways that regulate the function of tumor infiltrating lymphocytes [26-30]. Recent clinical trials of agents targeting PD-1 or PD-L1 have demonstrated durable tumor regression and prolonged stabilization of disease in patients with advanced non-small-cell lung carcinoma, melanoma, renal-cell carcinoma and Hodgkin's lymphoma [31-35].

In light of these findings, and with an interest in finding potential strategies for treating patients with high-grade meningioma, we have characterized the lymphocytic infiltrate of meningioma and demonstrate that there is an immunosuppressive microenvironment in anaplastic meningioma. Because the level of PD-L1 is the most important factor for predicting response to anti-PD-1 blockade [36] we have characterized PD-L1 expression using orthogonal techniques – immunohistochemistry and in situ hybridization with RNAscope [37]. Scoring of 291 cases separated into two cohorts using visual and several digital analysis platforms show that anaplastic meningioma have elevated PD-L1 protein and mRNA levels. This observation raises the possibility of testing immune checkpoint blockade for the immunotherapy of anaplastic meningioma.

## RESULTS

### Characterization of the immune infiltrate of meningioma in tissue specimens

**Figure 1:**
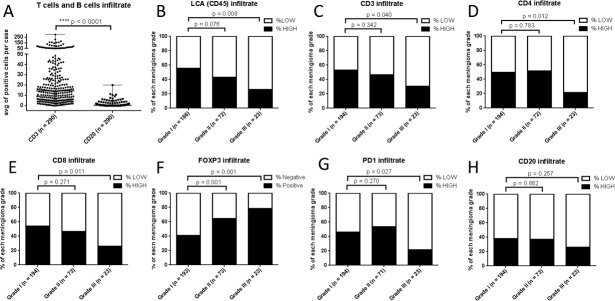
Characterization of immune cell infiltrate in meningioma A, Scatter plot of CD3+ T cells and CD20+ B cells in 290 cases of meningioma from TMAs 283 & 285 and TMA310. The percentage of cases with low or high infiltration (< or > the median score across all of the samples in the cohort for each marker, respectively) for B, LCA/CD45; C, CD3; D, CD4; E, CD8; F, FOXP3; G, PD-1; and H, CD20.

We characterized the lymphocytic infiltrate of meningioma in tissue resection specimens using three tissue microarrays containing samples from a total of 291cases – 195 WHO grade I, 73 WHO grade II and 23 WHO grade III (see Table S1 for subtype breakdown). We had constructed two of the TMAs (TMA283 & 285) previously for our study characterizing the mutation profile of meningioma [11]. We produced another TMA (TMA310) consisting of the 118 cases, most (n = 99) of which were diagnosed consecutively between 2012 and 2013 by the neuropathology service at Brigham and Women's Hospital. In addition to the standard meningioma subtypes (meningothelial, fibroblastic and transitional), these TMAs also contained cases of several less frequent subtypes (psammomatous, angiomatous, secretory and chordoid meningioma for instance). We stained each of the three TMAs with antibodies that recognize pan-lymphocyte marker (CD45/LCA), T-lymphocyte markers (CD3, CD4, CD8), B-cell marker (CD20), Treg marker (FOXP3) and immune regulatory marker PD-1 and quantified the data using both visual scoring by light microscopy review (Table S2) and Aperio Imagescope software (Fig. 1, Table 1 and Table S3). Notably, we found very similar results with both independent scoring methods (Table S2 and Table 1).

**Table 1:** Immune cell infiltrate in 291 cases of meningioma scored by Aperio digital algorithms (separated into low and high expressors - relative to the median score for each marker for the entire cohort; TMA 283 & 285 & 310)

Meningioma types	# of Cases	LCA			CD3			CD4		
		Low	High	Percent	Low	High	Percent	Low	High	Percent
WHO grade I	195	83	103	55.38%	91	103	53.09%	98	96	49.48%
Meningothelial	68	22	43		31	37		36	32	
Fibrous	46	25	20		22	24		29	17	
Transitional	52	20	31		22	30		23	29	
Angiomatous	11	6	4		6	5		4	7	
Psammomatous	9	3	3		3	5		2	6	
Secretory	9	7	2		7	2		4	5	
										
WHO grade II	73	41	31	43.06%	39	34	46.58%	35	37	51.39%
Atypical	68	36	31	p = 0.076	38	30	p = 0.342	32	35	p = 0.783
Chordoid	5	5	0		1	4		3	2	
										
WHO grade III	23	17	6	26.09%	16	7	30.43%	18	5	21.74%
Anaplastic	23	17	6	p = 0.008	16	7	p = 0.040	18	5	p = 0.012

CD8			CD20			FOXP3			PD-1		
Low	High	Percent	Low	High	Percent	Low	High	Percent	Low	High	Percent
89	105	54.12%	120	74	38.14%	114	79	40.93%	105	89	45.88%
29	39		38	30		42	26		30	38	
20	26		35	11		27	19		25	21	
21	31		33	19		26	25		29	23	
6	5		5	6		7	4		8	3	
5	3		5	3		5	3		5	3	
8	1		4	5		7	2		8	1	
											
39	34	46.58%	46	27	36.99%	26	47	64.38%	33	38	53.52%
37	31	p = 0.271	43	25	p = 0.862	24	44	p = 0.001	29	37	p =0.270
2	3		3	2		2	3		4	1	
											
17	6	26.09%	17	6	26.09%	5	18	78.26%	18	5	21.74%
17	6	p = 0.011	17	6	p =0.257	5	18	p = 0.001	18	5	p = 0.027

The numbers of LCA, CD3, CD4, CD8, CD20, and FOXP3 positive cells were counted using Aperio Imagescope software and the number of PD-1 positive cells was counted by visual microscopic review. The percentage of cases with low or high infiltration are presented for each marker (< or > the median score across all of the samples in the cohort for each marker, respectively).

We found that the lymphocyte infiltrate in the meningioma tissue was comprised predominantly of T cells (median = 14.3 CD3+ cells per core across all grades, Table S4) with infrequent B cells (median = 0.3 CD20+ cells per core across all grades, Table S4) (p < 0.0001) (Fig. 1A). We noted a significant decrease in the number of CD45/LCA+ cells in grade III meningioma samples (p = 0.008 comparing grade I with grade III) (Fig. 1B) with this decrease corresponding to a decrease in the number of CD3+ T lymphocytes (p = 0.040) (Fig. 1C) – including a decrease in both CD4+ lymphocytes (p = 0.012) (Fig. 1D) and CD8+ lymphocytes (p = 0.011) (Fig. 1E). To further characterize the lymphocyte infiltrate we stained the TMAs for the transcription factor FOXP3 that is a key transcription factor controlling T regulatory cell (Treg) development and function (Fig. 1F) as well as PD-1, an inhibitory cell surface receptor involved in the regulation of T-cell function (Fig. 1G). Interestingly, there was an increase in Foxp3+ cells in WHO grade II atypical meningioma and an even greater increase in WHO grade III anaplastic meningioma (Fig. 1F; p = 0.001). We also noted a drop in the number of PD-1+ cells in anaplastic meningioma, a change that was restricted to the anaplastic cases (Fig. 1G; p = 0.027). There was no significant difference in the number of B cells between the three grades of meningioma (Fig. 1H). There was a strong correlation between the number of CD45/LCA+ cells in each of the three cores from each case (Fig. S1A-C) suggesting limited heterogeneity in the samples and that the samples on the TMA are respresentative of the tumor sample.

### PD-L1 is expressed in meningioma cells

**Figure 2:**
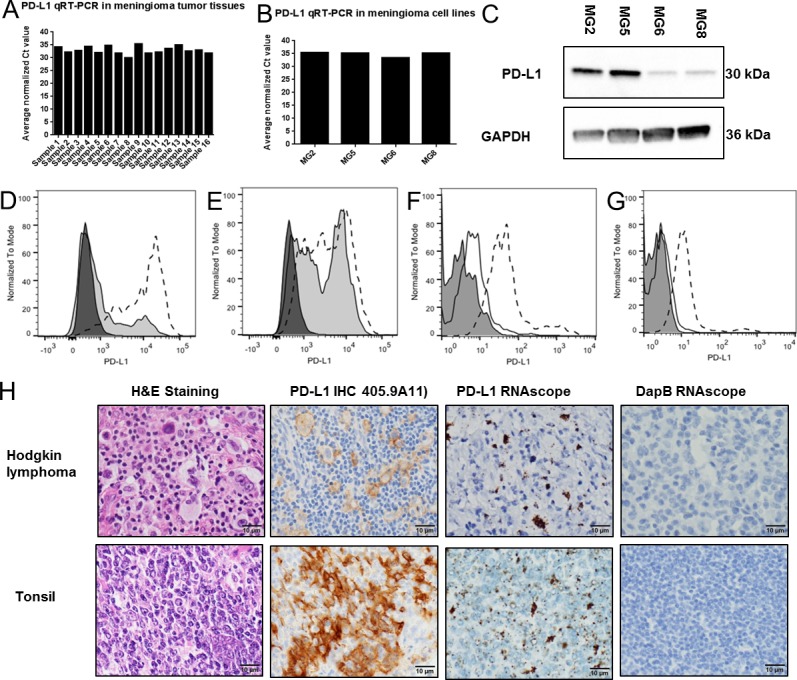
PD-L1 mRNA expression in meningioma tissues and cell lines A, PD-L1 RT-PCR in 16 meningioma frozen tumor resection tissues; B, PD-L1 RT-PCR in 4 patient derived meningioma cell lines (MG2, MG5, MG6 and MG8); C, PD-L1 and GAPDH immunoblots of lysates from 4 primary meningioma cell lines (MG2, MG5, MG6 and MG8); D, PD-L1 FACS analysis of CD45+CD33+ tumor infiltrating myeloid cells in two meningioma tissue suspensions (Shaded histogram, tumor M17. Dotted histogram, tumor M20); E, PD-L1 FACS analysis of the CD45− cell population, which is the population which includes the tumor cells, in two meningioma tissue suspensions (Shaded histogram, tumor M17. Dotted histogram, tumor M20); PD-L1 FACS analysis in two primary meningioma cell lines MG2 (F) and MG8 (G) (Filled histogram, unlabeled. line histogram, isotype control. Dotted histogram, PD-L1).H, In situ hybridization of PD-L1 mRNA transcripts using a PD-L1-specific RNAscope probe and immunhistochemistry of PD-L1 using a PD-L1 specific antibody (405.9A11) in sections from FFPE tissues blocks of Hodgkin's lymphoma (HL) and human tonsil.

The decrease in infiltrating T lymphocytes in anaplastic meningioma suggested to us that there is an immunosuppressive tumor microenvironment in these high-grade tumors. Because PD-L1 expression by tumor cells can mediate the inhibition of local immune responses, we next assessed whether meningioma cells express PD-L1 mRNA and protein. We detected the expression of PD-L1 mRNA in meningioma tumors using RT-PCR on RNA extracted from 16 frozen meningioma samples of various grades (Table S5). In all samples, we detected a specific signal for PD-L1 (Fig. 2A). Because the pattern of signal detected by RT-PCR in tissues cannot exclude that PD-L1 was expressed in infiltrating immune cells, we performed both RT-PCR (Fig. 2B) and immunoblot analysis (Fig. 2C) on four primary patient derived meningioma cell lines (MG2, 5, 6 and 8; Table S6) to determine if meningioma cells themselves express PD-L1. These cell lines had been cultured for 6 to 20 passages and therefore did not have residual lymphocytes. PD-L1 mRNA and protein was detected in each of these cell lines (Fig. 2B and 2C). The mRNA signal was substantially reduced following transfection of siRNA targeting PD-L1, supporting the specificity of the RT-PCR mRNA detection (Fig. S2A).

To confirm cell surface expression of PD-L1 on meningioma cells we next used FACS analysis. In two resections of atypical meningioma, we found that both CD45+CD33+ tumor infiltrating myeloid cells and the CD45− non-immune cells which include meningioma cells express PD-L1 (Fig. 2D and 2E). In addition, using two of the primary meningioma cell line cultures from the panel used for immunoblotting above (MG2 and MG8), we found that PD-L1 protein is expressed on the cell surface of these cells (Fig. 2F and G). A small subset (10% in MG2 and 5% in MG8) had detectably higher expression than the other cells in the population. Of note, PD-L1 mRNA was also detected in two additional primary meningioma cell lines (MG9 and MG10) and four established cell lines that are used in meningioma studies (Ben-Men-1, IOMM-Lee, F5 and CH157) (Fig. S2B).

To assess PD-L1 expression in tissue resection specimens and to correlate expression with meningioma grade in our TMA cohorts, we first developed and tested a custom-designed RNAscope probe (see Materials and Methods) for detecting human PD-L1 mRNA transcripts by in situ hybridization in FFPE tissues with 3,3′-Diaminobenzidine (DAB) used for signal visualization. A different fluorescence-labeled RNAscope probe for detecting PD-L1 was recently reported for the evaluation of breast cancer tissue [38]. With our probe we observed strong dot-like signals in cells from human tonsil and from Hodgkin lymphoma (HL) in which a subset of the cells are known to have high expression of PD-L1 (Fig. 2H). In the same tissues we saw no signal with a negative control DapB probe (Fig. 2H). In addition, the PD-L1 RNAscope did not show a signal in a range of normal human tissues including human kidney and liver and low-level signal in infrequent cells in the leptomeninges (Fig. S2C).

**Figure 3:**
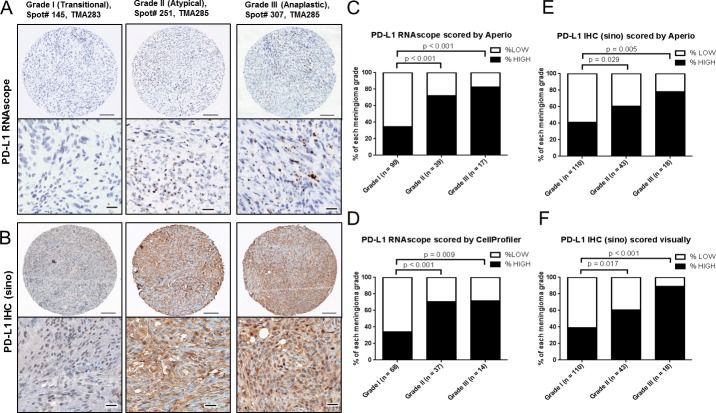
PD-L1 mRNA and protein expression in meningioma by grade A, Representative images of PD-L1 RNAscope staining and B, PD-L1 IHC Sinobiological antibody staining from WHO grade I (transitional), WHO grade II (atypical) and WHO grade III (anaplastic) meningioma from TMAs 283 & 285. Scale bar in low magnification, 100um; scale bar in high magnification, 20 um. PD-L1 RNAscope data was analyzed by C, Aperio and D, CellProfiler. PD-L1 IHC with a Sinobiological antibody was analyzed by E, Aperio and F, visual light microscopy review.

To confirm expression of PD-L1 in meningioma, we stained our three meningioma TMAs with the PD-L1 probe and detected signal in a portion of the samples – present at various levels in different cases (example images shown in Fig. 3A and S3). When expression was detected in a meningioma sample, signal was detected in most of the cells in the core, supporting that PD-L1 is expressed in meningioma cells (Fig. 3A and S3). Of note, 14 of the 16 frozen section samples examined by RT-PCR above had FFPE tissues in our TMAs and we observed a strong correlation (r = 0.5975; p = 0.0240) between the PD-L1 signal detected by RT-PCR and the signal detected by RNAscope PD-L1 probe scored by an Aperio pixel counting Imagescope software (Table S5 and Fig. S4A).

### PD-L1 expression is increased in WHO grade II and III anaplastic meningioma

**Figure 4:**
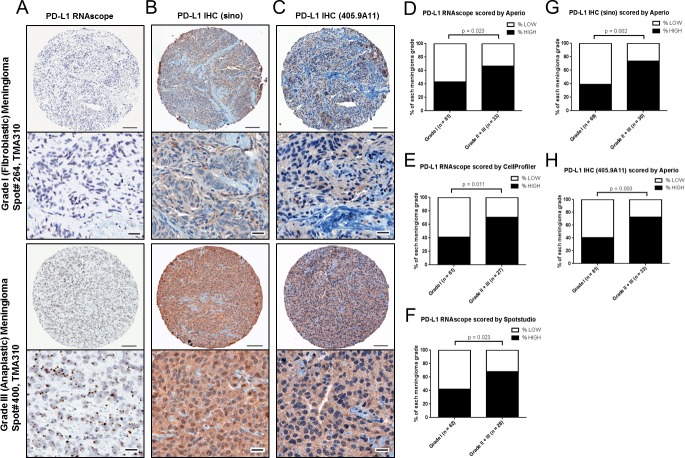
PD-L1 mRNA and protein expression in meningioma by grade in validation cohort A, Representative images of PD-L1 RNAscope staining, B, PD-L1 IHC (Sinobiological) antibody staining and C, PD-L1 IHC (405.9A11) antibody staining from a WHO grade I (fibroblastic) meningioma and from a WHO grade III (anaplastic) meningioma from a validation TMA (TMA310). Scale bar in low magnification, 100um; scale bar in high magnification, 20 um. PD-L1 RNAscope data was analyzed by D, Aperio and E, CellProfiler and F, Spotstudio™. Aperio pixel scoring was used to score PD-L1 IHC with the G, (Sinobiological) antibody and with the H, (405.9A11) antibody.

Having demonstrated that PD-L1 is expressed in meningioma tumors, we next proceeded to quantify the expression of PD-L1 mRNA (RNAscope) and protein (IHC) in our cohorts and investigated associations with WHO grade. We used the data from TMA283 & 285 as our investigation cohort (Cohort one) (Fig. 3) and then confirmed the results using the cases from TMA310 as a validation cohort (Cohort two) (Fig. 4). To rigorously evaluate our findings, we quantified mRNA signal from the discovery cohort (TMA283 & 285) using two digital analysis software – Aperio Imagescope which quantifies the number of positive pixels and intensity as well as the CellProfiler open source image quantification software [39] which permits cell by cell quantification of spot signals (dots per cell). To further validate our scoring algorithms we used Spotstudio™, a commercial software from the makers of RNAscope (Advanced Cell Diagnostics), to quantify the number of dots per cell in TMA310, in addition to Aperio Imagescope (pixel count) and CellProfiler (dot counting). Protein signal was quantified using a semi-quantitative assessment by visual review as well as using digital analysis software (Aperio Imagescope, positive pixel counting).

For RNAscope data analysis, in TMAs 283 & 285 there were a total of 173 meningioma cases (112 grade I, 43 grade II and 18 grade III). Fifteen cases had very low PPIB (which was used as a positive control) and were excluded from further analysis to avoid the possibility of false negative results. In total, 146 cases that were stained with our PD-L1 RNAscope probe could be evaluated by Aperio (Fig. 3C) and 119 cases by CellProfiler (Fig. 3D). By Aperio scoring (positive pixel counting), we found that compared to the expression in WHO grade I meningioma (median value = 102.5), there was significantly more PD-L1 mRNA expression in both grade II (median value = 291.3; p = 0.0012) and grade III (median value = 326.0; p = 0.0229) meningioma (Fig. S5A). Only 34.4% of WHO grade I meningiomas were PD-L1 mRNA high expressors (Fig. 3C) defined as cases with scores above the median for the entire cohort, 179.9, Table S4) whereas 71.8% of WHO grade II meningiomas were high expressors (p < 0.001) and 82.4% of WHO grade III meningiomas were high expressors (p < 0.001 (Fig. 3C). Scoring the numbers of signal spots per cell in the same two TMAs (TMA283 & 285) using the open source software CellProfiler analysis, we found similar results – that PD-L1 mRNA was highly expressed in 33.8% of WHO grade I, 70.3% WHO grade II, and 71.4% WHO grade III meningioma (Fig. 3D and S5B). The RNAscope Aperio (pixel counting) analysis and RNAscope CellProfiler (dot counting) analysis were highly correlated (r = 0.7652, p < 0.0001) (Fig. S6A).

To determine if PD-L1 mRNA levels correlate with PD-L1 protein levels we performed immunohistochemistry on TMA283 and 285 using a Sinobiological antibody (see Materials and Methods). For IHC staining, we observed a similar pattern of PD-L1 expression when we quantified protein expression by Aperio positive pixel counting (Fig. S5C) or by visual light microscopic scoring (Fig. S5D). Compared to the expression in WHO grade I meningioma (median value = 51.8), we noted significantly more PD-L1 protein expression in both WHO grade II (median value = 96.7; p < 0.0001) and WHO grade III (median value = 83.3; p = 0.0010) meningioma (Fig. S5C). Only 40.9% of WHO grade I meningioma were PD-L1 protein high expressors (defined as cases with scores above the median of 63.1 for the entire cohort; Table S4) whereas 60.5% of WHO grade II meningioma were high expressors (p = 0.029) and 77.8% (14 of 18 cases) of WHO grade III meningiomas were high expressors (p = 0.005) (Fig. 3E). Similar results were observed when the IHC stained slides were scored by visual review with high PD-L1 protein expression in 39.1% WHO grade I, 60.5% WHO grade II, and 88.9% WHO grade III meningioma (Fig. 3F). The IHC Aperio analysis and visual analysis were highly correlated (r = 0.8276, p < 0.0001) (Fig. S6B). We noted a non-linear correlation between mRNA expression (RNAscope Aperio) and protein expression (by IHC (sino) Aperio or by IHC (sino) visual) in this TMA cohort (r = 0.4890, p < 0.0001; r = 0.3805, p < 0.0001) (Fig. S6C and S6D).

To validate the strong association between WHO meningioma grade and both PD-L1 mRNA and protein expression we used a separate cohort (TMA310), and analyzed staining of the same RNAscope probe (Fig. 4A) and two different IHC antibodies – the same PD-L1 Sinobiological antibody used for IHC of TMAs 283 & 285 (Fig. 4B) and an additional PD-L1 antibody (405.9A11) (Fig. 4C). In TMA310 there were a total of 118 meningioma cases (83 grade I, 30 grade II and 5 grade III). Because this cohort is comprised of consecutive clinical cases over the last two years at Brigham and Women's Hospital and therefore reflects the frequency of cases in active clinical practice, only 5 of the 118 cases are WHO grade III anaplastic meningioma. So, for the analysis we grouped WHO grade II and III meningioma together as a “high-grade” group. Notably, the results in this cohort are very similar to those we observed with TMAs 283 & 285.

For RNAscope data analysis, 2 cases with very low PPIB were excluded from further analysis. For the Aperio analysis of PD-L1 RNAscope data 114 cases each having at least one informative core were available (Fig. S5E), for CellProfiler, 88 cases were available (Fig. S5F) and for Spotstudio™ 90 cases were available for analysis (Fig. S5G). The Aperio PD-L1 RNAscope (pixel counting) analysis revealed that compared to the expression in WHO grade I meningioma (median value = 142.0), there was significantly more PD-L1 mRNA expressed in high-grade meningioma (median value = 355.0; p = 0.0275; (Fig. S5E). Only 43.2% of the WHO grade I meningioma were PD-L1 mRNA high expressors whereas 66.7% of the high-grade meningioma were high expressors (p = 0.023; Fig. 4D). The CellProfiler PD-L1 RNAscope (dot counting) analysis showed that PD-L1 mRNA was highly expressed in 41.0% of WHO grade I meningioma and in 70.4% of high-grade meningioma (Fig. 4E). Consistent with the results from both Aperio analysis and CellProfiler analysis (Fig. S5E and S5F), the analysis using Spotstudio™ (dot counting) software (Fig. S5G) showed that PD-L1 mRNA was highly expressed in 41.9% of WHO grade I meningioma and in 67.9% of WHO grade II and III meningioma (Fig. 4F). There was very high correlation between the three methods (Aperio, CellProfiler and Spotstudio™) that we used to score PD-L1 mRNA expression even though we measured staining intensity with pixel counts with Aperio Imagescope and individual dots per cell using CellProfiler and Spotstudio (Fig. S6E,6F, S6G; r values between 0.7839 and 0.9105). Moreover, 12 of the cases that had frozen samples available and that we had previously analyzed with RT-PCR also showed strong correlation between the RT-PCR values of PD-L1 and the scoring of PD-L1 RNAscope by Aperio (r = 0.5975), by CellProfiler (r = 0.7374) and by Spotstudio™ (r = 0.7033) (Fig. S4 and Table S5). Similar to the limited heterogeneity seen for CD45/LCA+ cells, variability of PD-L1 mRNA expression between cores was low with high correlations between the three cores from each case (Fig. S1D-F).

Similar to the protein expression results observed in TMAs 283 & 285, we noted a strong correlation between PD-L1 protein expression and WHO meningioma grade in the TMA310 validation cases. For this analysis we used two antibodies that recognize PD-L1 – PD-L1 (Sinobiological) (Fig. 4B) and PD-L1 (405.9A11) (Fig. 4C) antibodies and scored the staining using Aperio Imagescope software (Fig. S5H and S5I). For the PD-L1 Sinobiological antibody, we observed that PD-L1 protein was highly expressed in 39.1% of WHO grade I meningioma but was highly expressed in 73.3% of higher grade meningioma (Fig. 4G and S5H). The PD-L1 (405.9A11) antibody showed similar results, with PD-L1 protein highly expressed in 40.7% of WHO grade I meningioma and in 72.7% of higher meningioma (Fig. 4H and S5I). The staining by these two antibodies was significantly correlated (r = 0.6152, p < 0.0001) (Fig. S6H). We also noted a non-linear correlation between mRNA expression (RNAscope Aperio) and protein expression (by IHC with both PD-L1 (sino) and PD-L1 (405.9A11) antibodies) (Fig. S6I and S6J). Expression of PD-L1 mRNA and protein were not associated with a history of prior radiation therapy (237 patients with no history of radiation therapy and 40 with a history of prior radiation therapy).

### PD-L1 expression is not independently associated with outcome

We performed cox proportional hazards modeling to investigate the impact of PD-L1 expression levels and grade on time to progression and all-cause mortality (in cohort one and two combined), and also assessed other clinical variable that have shown associations with grade such as age, performance status, extent of resection, and use of RT. On univariate analysis, grade III was highly correlated with both shorter time to progression (HR = 5.85, p < 0.0001) and all-cause mortality (HR = 2.64, p = 0.02). Additional variables associated with time to progression were age at diagnosis (HR = 1.03 per year increase, p = 0.01), and gross total resection (HR = 0.31, p=0.0005). Additional variables associated with all-cause mortality included only gross total resection (HR = 0.31, p=0.0006). After adjusting for grade, extent of surgical resection, and receipt of radiation therapy, neither Aperio pixel level or Cellprofiler score as associated with time to progression (p = NS). Similarly, after adjusting for grade, neither Aperio pixel level or CellProfiler score as associated with all-cause mortality (p = NS) suggesting that while PD-L1 may be a biomarker that may guide therapeutic intervention, that it is not, in this cohort, an independent prognostic indicator of poor outcome.

## DISCUSSION

Our results demonstrate an alteration of the immune infiltrate of meningioma as these tumors become increasingly aggressive. In particular, we note a significant decrease in CD4+ and CD8+ T lymphocytes, a decrease in PD-1+ lymphocytes and an increase in FOXP3+ Treg lymphocytes in WHO grade III meningioma. Such a shift in the immune profile suggests that immune modulatory mechanisms may be important for the progression of meningioma and for the malignant behavior characteristic of WHO grade III meningioma. Notably, using several orthogonal analytical approaches we observe a significant increase in the expression of the mRNA and protein levels of the clinically relevant and actionable immune regulatory molecule PD-L1 in WHO grade III meningioma.

The pronounced clinical responses to immune blockade observed in other solid tumors such as advanced non-small-cell lung carcinoma, melanoma, and renal-cell carcinoma raises the possibility that testing of immune blockade strategies may be warranted in patients with aggressive high grade meningiomas that are associated with a poor clinical prognosis. It is reasonable to consider the work we present in this manuscript as hypothesis generating and that further validation in other cohorts will be optimal to determine that our analysis was not biased and that the results are generalizable. Prospective studies will be needed to validate the ability to assay PD-L1 as a biomarker in real time and to determine if there is an association between the level of PD-L1 expression and response to treatment. Our data suggests that while PD-L1 expression is increased in higher grade meningioma, that it itself is not an independent prognostic indicator of outcome. Future studies with a larger number of samples and longer follow up will be needed to confirm this observation.

Prior studies of the immune infiltrate in meningiomas have focused predominantly on investigating the monocytic infiltrate of these tumors [40, 41] while others have characterized B and T lymphocyte infiltrates but on small sample sets [42, 43] (less than 30 samples). One study used flow cytometry analysis to characterize the lymphocyte population infiltrating 28 cases of meningioma [42] and demonstrated several important findings: that meningioma harbor both T and B lymphocytes that are antigen-experienced; that meningioma have CD4+ and CD8+ memory/effector T cells, regulatory T cells, and T cells expressing the immune checkpoint molecules PD-1; that these effector T cell populations are enriched relative to peripheral blood suggesting that certain T lymphocytes migrate to and reside in the tumors; and that the T lymphocytes infiltrating meningioma show phenotypes associated with ‘exhaustion’ suggesting that they may not be able to mount an effective immune response. Because this study used flow cytometry to characterize fresh meningioma tissues, the numbers of samples analyzed was relatively small (including only three WHO grade III meningiomas) compared to studies that use retrospective cohorts of FFPE fixed tissues. Hence, the authors could not assess if there are differences in the immune cell infiltrating populations of meningioma according to WHO grade and if there are significant changes in the expression of immune modulatory molecules in meningioma, particularly in the meningioma cells themselves. Our study, utilizing nearly 300 FFPE meningioma samples allowed us to address this important clinical issue by characterizing large numbers of samples and assessing the differences in the tumor infiltrating immune cells in high grade meningioma, the cases that are most likely to require treatment. A limitation of TMA based studies, on the other hand, is that the extracted TMA cores may miss potential heterogeneity that is present in the patient samples. Because we see high correlation between the scores of different cores for each patient samples, this suggests that the heterogeneity in meningioma may be less than that seen in other tumors, consistent with their relatively uniform morphological appearance seen by light microscopy.

Because antibodies for recognizing PD-L1 have some reported limitations [38, 44, 45], we developed a custom-designed RNAscope probe for detecting human PD-L1 mRNA transcripts. We thought that such a probe would have distinct advantages such as reduced background and increased specificity as well as readily quantifiable results. Indeed, using this probe we observed signal only in tissues known to harbor PD-L1 expressing cells like tonsil and Hodgkin's lymphoma and not in several other normal human tissues. Notably, even though we observed a significant correlation between PD-L1 mRNA expression as detected by RNAscope in situ hybridization and PD-L1 protein expression as detected by IHC using two different anti-PD-L1 antibodies, the signal to noise ratio of the RNAscope probe was far better than that observed with IHC detection. This suggests that it may be important to test PD-L1 in situ hybridization probes alongside antibodies in clinical trial settings, especially in cohorts that are powered to capture correlations between clinical responses and PD-L1 expression levels. Such probes for immune regulatory molecules may become important biomarkers for predicting response to treatment.

Recent studies demonstrated the use of a different fluorescence-labeled RNAscope probe for detecting PD-L1 in tumor tissues [38]. The DAB visualized probe that we used offers the advantage of readily correlating RNA expression with underlying histology using techniques and approaches that are customary to surgical pathologists. While proprietary algorithms have been streamlined to facilitate analysis of the RNAscope signals, we provide and validate an open-source CellProfiler image analysis software pipeline that can be readily optimized and implemented for quantifying RNAscope signals. This freely available software analysis pipeline could encourage the increased utilization of RNAscope in situ hybridization testing by research and clinical laboratories alike and could therefore facilitate the incorporation of RNAscope technologies into routine digital pathology workflows as well as into clinical trial correlative analyses.

## MATERIALS AND METHODS

### Patient cohorts, clinical tissue samples, tissue microarrays and cell lines

For this retrospective study, we used two sets of archival human meningioma formalin-fixed, paraffin-embedded (FFPE) tissue specimens that we had collected from the Department of Pathology, Brigham and Women's Hospital, Harvard Medical School, Boston, Massachusetts. The study was approved by the Institutional Review Boards (IRBs) of Brigham and Women's Hospital and Dana Farber Cancer Institute, Harvard Medical School. Details are available in in the Supplemental Materials and Methods.

### RNAscope assay and evaluation

In situ detection of PD-L1 transcripts in FFPE TMA samples was performed using the RNAscope® assay with Probe-Hs-PDL1-v2 (Cat# 409671, Advanced Cell Diagnostics, USA) and RNAscope® 2.0 HD Reagent Kit (BROWN) (Cat# 310035, Advanced Cell Diagnostics, USA) following the protocols suggested by the manufacturer. See Supplemental Materials and Methods for details on staining and scoring with Aperio Imagescope, Spotstudio and Cell profiler.

### Immunohistochemistry and qRT-PCR assay

Immunohistochemistry (IHC) was performed using a BOND III staining system (Leica Microsystems). The following primary antibodies were used in this study: LCA (1:600 dilution, Cat# M0701; Dako, CA), CD3 (1:250 dilution, Cat# A0452; Dako, CA), CD4 (1:80 dilution, Cat# M7310; Dako, CA), CD8 (1:100 dilution, Cat# M7103; Dako, CA), CD20 (Ready to Use, Cat# N1502 RTU; Dako, CA), FOXP3 (1:50 dilution, Cat# 320102; Biolegend, CA), PD-1 (1:300 dilution, Cat# 315M-95; Cell Marque, CA), EMA (1:100 dilution, clone: E29, Cat# M0613; DakoCytomation, CA), PD-L1 (1:36 dilution, Cat# 10084-R015; Sinobiological, China), PD-L1 (1:125 dilution, 405.9A11; courtesy of Gordan Freeman lab DFCI) [46]. The staining protocols and scoring and details on the qRT-PCR are reported in the Supplemental Materials and Methods.

### Immunoblot

Meningioma cell lines were harvested and lysed with RIPA buffer (Thermo Scientific, USA) and halt Protease Inhibitor Cocktail (Thermo Scientific, USA). Equal amounts of denatured protein sample were separated by SDS-PAGE and were then transferred to PVDF membranes using Trans-Blot® Turbo™ Midi PVDF Transfer Packs (Bio-Rad, USA) for immunoblot analysis. We used antibodies to recognize PD-L1 (1:200 dilution, Cat# ab58810, Abcam, USA) [47, 48], GAPDH (1:500 dilution, Cat# sc-32233, Santa Cruz, USA) as loading control. All protein bands were detected using Western Blotting Luminol Reagent (Cat# sc-2048, Santa Cruz, USA).

### Fluorescence Activated Cell Sorting (FACS) Analysis

Cryo-preserved meningioma single-cell suspensions were thawed at room temperature. The tumor suspensions were incubated on ice with anti-biotin microbeads (Miltenyi) and run through a magnetic column to remove excess debris from the cell slurry. The cells were split and stained with fluorochrome-labeled mAbs for CD33 (WM53, BD Biosciences), CD45 (HI30, BioLegend, PD-L1 (29E.2A3, Biolegend) or an isotype control (mouse IgG2b, BioLegend) and fixed in 4% paraformaldehyde. Samples were run on either a FACSCalibur™ or FACSAria™ II (BD Biosciences) with CellQuest software and analyzed using FlowJo software (Tree Star).

### SiRNA transfection and Hematoxylin and Eosin (H&E) staining

Detailed information of siRNA transfection and (H&E) staining are provided in the Supplemental Materials and Methods.

### Statistical analysis

Details of analysis are provided in the Supplemental Materials and Methods.

## SUPPLEMENTARY METHODS FIGURES AND TABLES














