# Supplementary material for: Increased expression of the immune modulatory molecule PD-L1 (CD274) in anaplastic meningioma
Source: Oncotarget. 2014 Dec 31;6(7):4704–16. doi: 10.18632/oncotarget.3082 (PMC4467109; doi:10.18632/oncotarget.3082)
Supplement: Supplementary file 1 [file oncotarget-06-4704-s001.pdf]

Increased expression of the immune modulatory molecule PD-L1 (CD274) in anaplastic meningioma

Supplementary Material

Materials and Methods

Patient cohorts, clinical tissue samples, tissue microarrays and cell lines

For this retrospective study, we used two sets of archival human meningioma formalin-fixed, paraffin-embedded (FFPE) tissue specimens that we had collected from the Department of Pathology, Brigham and Women's Hospital, Harvard Medical School, Boston, Massachusetts. The samples were compiled in tissue microarray (TMA) format and organized in investigational (cohort one) and validation (cohort two) cohorts. Cohort one (n = 173 cases) was comprised of samples from two previously described TMAs (TMA283 & TMA285) [1]. Cohort two included 118 meningioma resection samples compiled in a separate TMA we compiled in a single TMA (TMA310). Ninety-nine of these of these cases were diagnosed consecutively between 2012 to 2013 on the neuropathology service at Brigham and Women's Hospital. We collected clinicopathological information for these cases from clinical records and pathology reports (**Table S1**). Two trained neuropathologists (MA and SS) selected representative regions of tumors by microscopic review of Hematoxylin and Eosin (H&E) stained sections and three 0.6 mm cores were taken from each of these regions of interest from each tumor and arrayed in recipient blocks. We collected FFPE tissues of Hodgkin lymphoma (HL), human tonsil, human liver and human kidney from the Department of Pathology, Brigham and Women's Hospital, Harvard Medical School, Boston, Massachusetts.

For the qRT-PCR assay, we obtained 16 unfixed, frozen meningioma tissue samples from the Brigham and Women's Hospital Tumor Bank (see Results for details).

Primary human meningioma cell lines MG2, MG5, MG6, MG8, MG9 and MG10 were cultured in Stemedia™ WIT-T™ Culture Media (Cat# 00-0047-500, STEMGENT, California, United States) containing 20% fetal bovine serum and 1% penicillin/ streptomycin solution (see Results for details about cell lines). The established human meningioma cell lines Ben-Men-1, F5, IOMM-Lee and CH157 were from Dr. Ian Dunn's lab in the Department of Neurosurgery, Brigham and Women's Hospital, Harvard Medical School, Boston, Massachusetts. The cell lines are not available from commercial cell banks and authentication was not performed. All the cell lines were grown in a humidified incubator at 37°C with 5% CO_2.

The study was approved by the Institutional Review Boards (IRBs) of Brigham and Women's Hospital and Dana Farber Cancer Institute, Harvard Medical School.

RNAscope assay and evaluation

5 um FFPE sections were deparaffinized, boiled with pretreatment reagent for 15 minutes, and then protease digested at 40ºC for 30 minutes, followed by hybridization for 2 hours at 40ºC with Probe-Hs-PDL1-v2. The probe was visualized with 3,3'-Diaminobenzidine (DAB) and cell nuclei were stained with Hematoxylin. The Probe-DapB (Cat# 310043, Advanced Cell Diagnostics, USA) was used as a negative control, and Probe-Hs-PPIB (Cat# 313901, Advanced Cell Diagnostics, USA) was used as a positive control. All the slides were digitally scanned, then visually evaluated by two pathologists (ZD and SS). We excluded cases from particular analyses if there were staining artifacts, less than 90% tumor remaining in the cores or if there were very low or undetectable levels of PPIB.

Aperio pixel counting: We optimized the Aperio Pixels Algorithm (Aperio, Leica Biosystems) to count positive pixels (total stain, not individual dots) from PD-L1 RNAscope® staining of TMA283 & 285 and TMA310 slides. We determined the average pixels value for each case as the mean value of the available cores per case (one to three). For analysis, we classified all cases with average pixel values greater than the median for all cases in a cohort as high PD-L1 expressors, and we classified all cases with average pixel values lower than the median for all cases in a cohort as low PD-L1 expressors.

Spotstudio™ counting: We counted the number of dots from PD-L1 RNAscope® stained TMA310 slides using proprietary commercial software – Spotstudio™ –from Advanced Cell Diagnostics, USA. Similar to the analysis for Aperio pixel counting, we classified all cases with an average number of dots per cell that was greater than the median for all cases in a cohort as high PD-L1 expressors, and we classified all cases with an average number of dots per cell that was lower than the median for all cases in a cohort as low PD-L1 expressors.

CellProfiler counting: We also used the open-source CellProfiler image analysis software (http://www.cellprofiler.org/) to analyze the number of dots from PD-L1 RNAscope® of TMA283 & 285 and TMA310 slides. We developed an analysis pipeline that included the following modules: the 'UnmixColors' module was used to split each image into 1) Hematoxylin stain and 2) DAB stain images, and then cell nuclei and cell cytoplasm were identified using the Hematoxylin stain images. Next, the 'EnhanceOrSuppressFeatures' module was used to remove background and highlight and count the spots in the DAB stain image. Finally the 'RelateObjects' module established which spots belong to which cells, and spots per-cell were counted. The pipeline was optimized for meningioma TMAs, and is available online (http://www.cellprofiler.org/published_pipelines.shtml). Optimization for scoring other tumor types will require optimizing diameter parameters for detecting nuclei and for detecting in situ hybridization signals (dots). Similar to the analysis for Aperio pixel counting, we classified all cases with an average number of dots per cell that was greater than the median for all cases in a cohort as high PD-L1 expressors, and we classified all cases with an average number of dots per cell that was lower than the median for all cases in a cohort as low PD-L1 expressors.

For TMA310, the Aperio Imagescope software recognition of the staining signal was of high quality in 114 cases, but improper detection of spots was higher with CellProfiler and Spotstudio, requiring a subset of cases to be excluded from the analyses - 88 cases were scored with CellProfiler (26 cases were excluded) and 90 with Spotstudio (24 cases were excluded). 87 cases were scored with both CellProfiler and Spotstudio indicating that similar cases were excluded using both image analysis systems. Visual review of problematic cases revealed false positive recognition of stromal cells such as blood cells in regions of hemorrhage as well as dark staining of some nuclei by the counterstain. Further optimization of staining parameters may be needed in future TMA-based studies; possibly such artifacts will not be problematic when whole stained sections are available for review.

Immunohistochemistry and evaluation

The following primary antibodies were used in this study: LCA (1:600 dilution, Cat# M0701; Dako, CA), CD3 (1:250 dilution, Cat# A0452; Dako, CA), CD4 (1:80 dilution, Cat# M7310; Dako, CA), CD8 (1:100 dilution, Cat# M7103; Dako, CA), CD20 (Ready to Use, Cat# N1502 RTU; Dako, CA), FOXP3 (1:50 dilution, Cat# 320102; Biolegend, CA), PD-1 (1:300 dilution, Cat# 315M-95; Cell Marque, CA), PD-L1 (1:36 dilution, Cat# 10084-R015; Sinobiological, China), PD-L1 (1:125 dilution, Cat# 405.9A11; courtesy of Gordan Freeman lab DFCI). We used 3,3'-Diaminobenzidine (DAB) for visualization. We substituted non-immune goat or rabbit serum for the primary antibodies for negative controls. We used an Aperio scanner to acquire digital images from all stained TMA slides. Digital images were scored using Aperio ImageScope software. We also had a study pathologist (ZD) evaluate stains by light microscopy while blinded to clinical and pathologic information using semi-quantitative scoring as described below.

For characterizing the immune infiltrate of meningioma in TMA283 & 285 and TMA310, we counted LCA, CD3, CD4, CD8, CD20 and FOXP3 positive cells using an optimized Aperio pixel counting algorithm (Aperio, Leica Biosystems). We assigned the mean value for each marker for all available cores as the average positive cells for each case. We classified all cases with average mean values greater than the median for all cases in a cohort as high expressors of a particular marker, and we classified all cases with average mean values lower than the median for all cases in a cohort as low expressors of a particular marker.

In addition, we counted LCA, CD3, CD4, CD8, CD20, FOXP3 and PD-1 positive cells visually by light microscopy in TMA 283 & 285 as well as the PD-1 positive cells in TMA310. For LCA, CD3, CD4, CD8 and CD20 we considered average positive cells greater than or equal to 10 as ‘high infiltrate’, and average positive cells less than 10 as ‘low infiltrate.’ For FOXP3 and PD-1

positive cells, the average positive cells greater than or equal to 1 per case was considered as ‘high infiltrate,’ and average positive cells less than 1 was considered as ‘low infiltrate.’

For PD-L1 IHC quantification, we determined an H-score by optimized Aperio pixel counting algorithm (Aperio, Leica Biosystems). The average H-score for each case was the mean value of available cores. An average H-score greater than the median for all cases was considered as ‘high expression,’ and average H-score less than or equal to the median for all cases value was considered as ‘low expression.’

In addition, we semiquantitatively estimated by visual microscopic review PD-L1 IHC staining using a composite score obtained by multiplying the values of staining intensity and relative abundance of positive cells. Intensity was graded as 0 (no staining), 1 (weak staining), 2 (moderate staining), or 3 (strong staining). The abundance of positive cells was graded from 0 to 4 (0, <5% positive cells; 1, 5–25%; 2, >25–50%; 3, >50–75%; 4, >75%). A composite score greater than the median value for all cases was considered ‘high expression,’ and composite score less than or equal to the median value for all cases was considered ‘low expression.’

qRT-PCR assays

For qRT-PCR assay, we extracted total RNA from frozen meningioma tissues and cell lines using TRIzol Plus RNA Purification Kit (Cat# 12183-555, Life Technologies) according to the manufacturer’s protocols and we used high capacity cDNA Reverse Transcription Kit with Rnase Inhibitor (Cat# 4374966, Life Technologies) for reverse transcription. We performed PD-L1 qRT-PCR detection using a TaqMan primer/probe set for CD274 (Hs01125301_m1) (Cat# 4331182, Life Technologies) and TaqMan® Universal Master Mix II, with UNG (Cat# 4440042, Life Technologies). We used as an internal control eukaryotic 18S rRNA Endogenous Control (Cat# 4333760T, Life Technologies). The PD-L1 Ct value was normalized to 18S rRNA Ct value, and the average normalized PD-L1 Ct value is presented. Moreover the relative expression level was determined as $2^{-\Delta\Delta Ct}$, and data is presented as the expression level relative to the calibrator (control sample).

siRNA transfection

Before transfection, 2×105cells per well were plated into 6-well plates and grown for one day. When the cell confluent was reached to 40% to 60%, cells were transfected with PD-L1 specific siRNAs pool and negative control siRNA (sigma, USA) by using lipofectamine RNAiMAX reagent (Cat# 13778-030, Life technologies, USA), Transfected cells were grown at 37℃ for 6 h, followed by incubation with complete medium. The cells were harvested for RNA extraction at 48 h post-transfection.

Hematoxylin and Eosin (H&E) staining

H&E staining was performed in clinical lab at Department of Pathology, Brigham and Women's Hospital using standard procedure.

Statistical analysis

We used the Chi-Square test to evaluate the differences between high and low expressors of LCA, CD3, CD4, CD8, CD20, FOXP3 and PD1 positive cells, of RNAscope® Aperio pixel count values, of RNAscope® CellProfiler values, of RNAscope® Spotstudio™ values, and of IHC Aperio values between the different grades of meningioma.

We used the unpaired t test to evaluate the differences between the numbers of T cells (CD3-positive) and B cells (CD20-positive) infiltrating meningioma, the RNAscope® Aperio values, the RNAscope® CellProfiler values, the RNAscope® Spotstudio™ values and the IHC Aperio values in different grades of meningioma. We also evaluated the correlations between RNAscope® Aperio values, RNAscope® CellProfiler values, RNAscope® Spotstudio™ values, IHC Aperio values. We considered p values of < 0.05 as statistically significant. We analyzed data using Graphpad Prism 6 (unpaired t test, and correlation analysis) and SPSS 12.0 (Chi-Square test).

Cox proportional hazards modeling was used to assess the impact of PD-L1 expression levels on time to progression and all-cause mortality in meningioma . Time to progression was defined as the time from pathologic diagnosis date to salvage therapy, serial radiographic progression, or death, censored at the time of last MRI assessment. All-cause mortality was defined as time of pathologic diagnosis to death, censored for last clinical encounter. In addition to PD-L1 expression and grade, clinical variables that have shown associations with these endpoints such as age, performance status, extent of resection, and use of RT were included in the analysis. Statistical analyses were performed using SAS v9.3 (SAS Institute, Cary, NC).

References:

1. Brastianos PK, Horowitz PM, Santagata S, et al. Genomic sequencing of meningiomas identifies oncogenic SMO and AKT1 mutations. *Nature genetics.* 2013; 45(3):285-289.

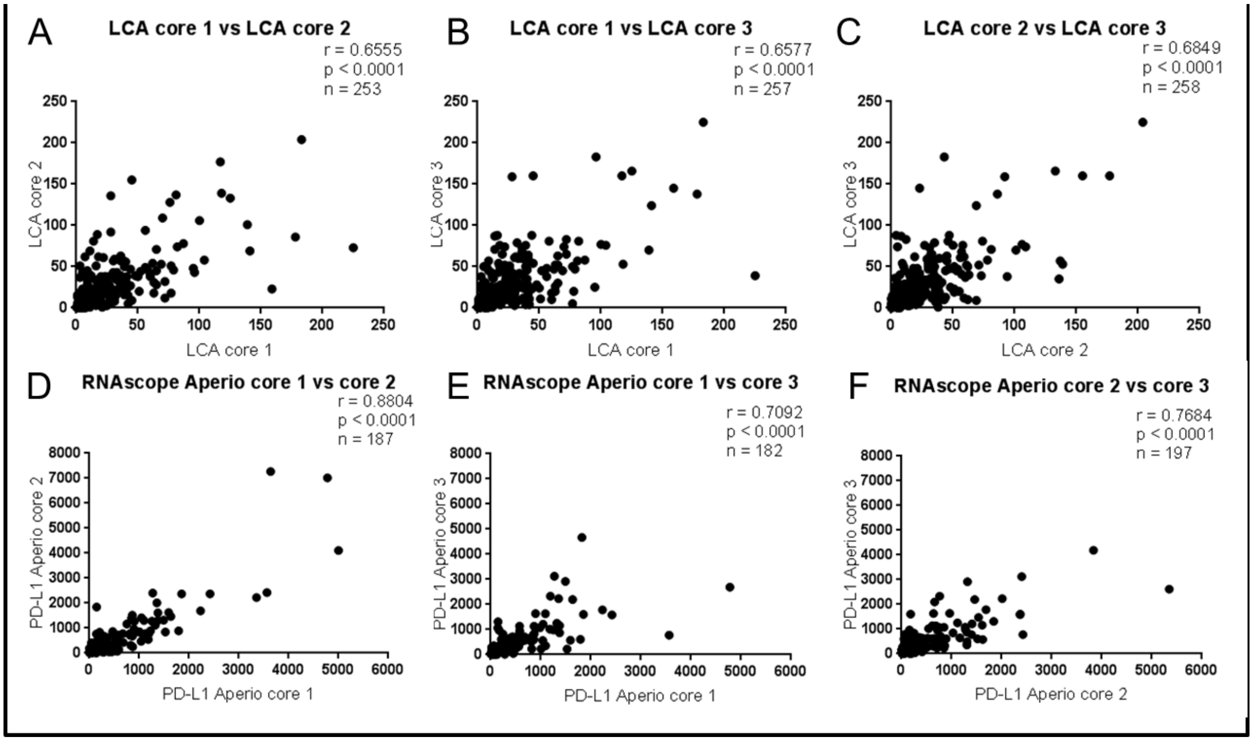

Figure S1: Correlation between scores of cores on TMAs. A-C, Correlation analysis of immunohistochemistry for CD45/LCA+ cells between groups of cores (cores 1, 2 and 3) on TMA 283, 285 and 310. D-E, Correlation analysis of PD-L1 RNAscope scored by Aperio Imagescope between groups of cores (cores 1, 2 and 3) on TMA 283, 285 and 310.

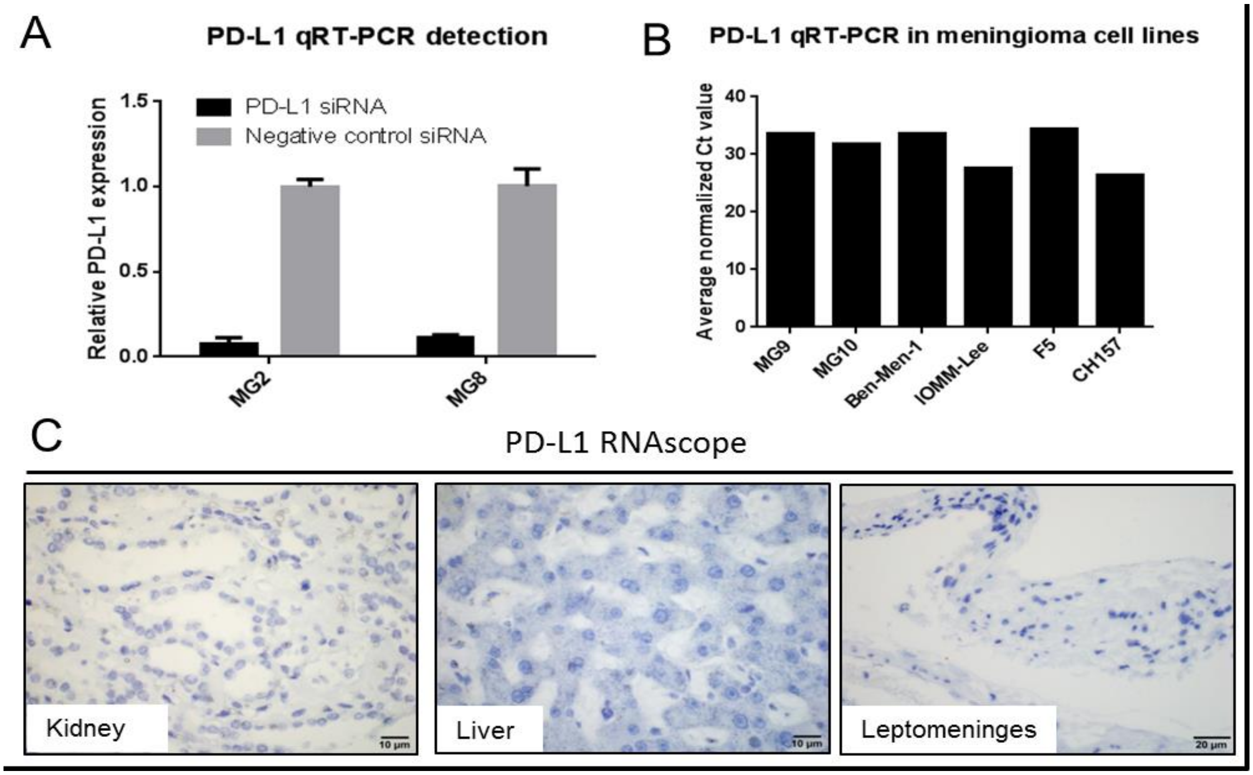

Figure S2: PD-L1 mRNA expression in meningioma cell lines and leptomeninges. A, PD-L1 RT-PCR in PD-L1 siRNA pool treated MG2 and MG8 cells; B, PD-L1 RT-PCR in a panel of patient derived meningioma cell lines (MG9 and MG10) as well as established cell lines used in meningioma research (Ben-Men-1, IOMM-Lee, F5 and CH157). C, In situ hybridization of PD-L1 mRNA transcripts using a PD-L1-specific RNAscope probe visualized with 3,3'-Diaminobenzidine (DAB) in sections from FFPE tissues blocks of human kidney, human liver and human leptomeninges.

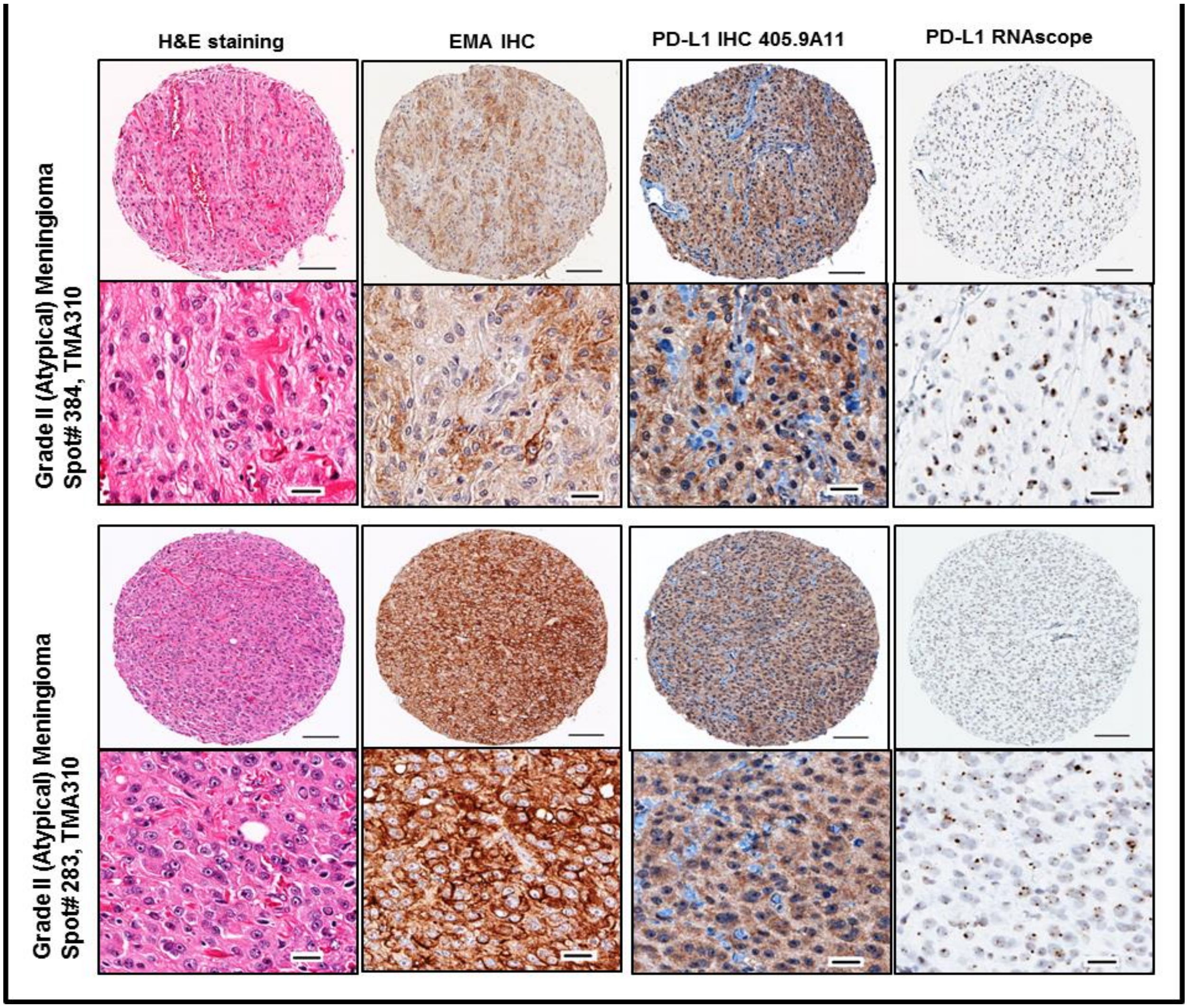

Figure S3: PD-L1 expression in meningioma. Representatitive images from H&E staining, EMA IHC, PD-L1 IHC (405.9A11) and PD-L1 RNAscope from TMA310.

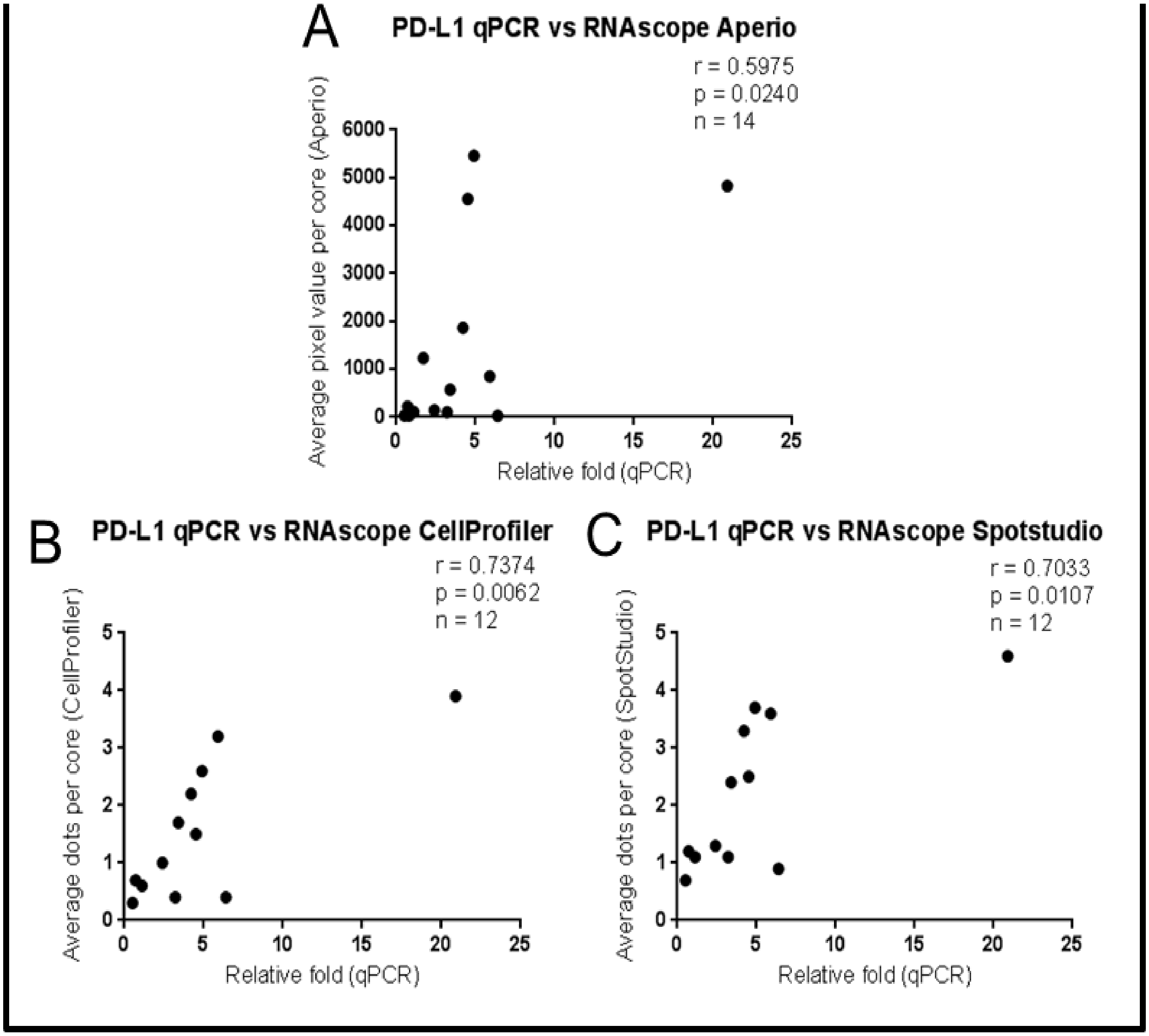

Figure S4: Correlation analysis of PD-L1 RT-PCR using RNA from frozen meningioma specimens and PD-L1 mRNA detected by RNAscope in situ hybridization using corresponding FFPE samples from the same cases that were scored by Aperio, Spotstudio™ and CellProfiler. Numbers of cases analyzed is indicated.

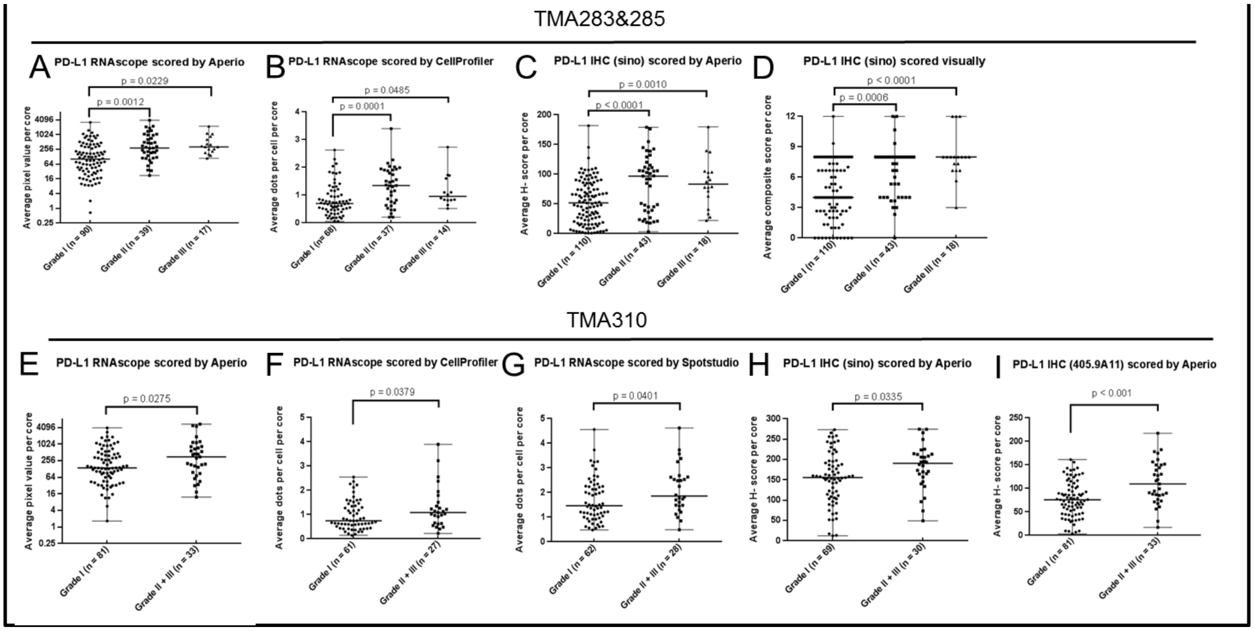

Figure S5: PD-L1 mRNA and protein expression in meningioma by grade. Scatter plot representations of PD-L1 mRNA expression in cases from TMAs 283 & 285 as detected by RNAscope in situ hybridization and scored by A, Aperio pixel counting with Imagescope software, and B, CellProfiler open source image analysis software and scatter plot representations of PD-L1 protein expression as detected by IHC using a Sinobiological antibody recognizing PD-L1 and scored by C, Aperio pixel counting with Imagescope software and by D, visual light microscopy review. Scatter plot representations of PD-L1 mRNA expression in cases from TMA310 as detected by RNAscope in situ hybridization and scored by E, Aperio pixel counting with Imagescope software, F, CellProfiler open source image analysis software and G, Spotstudio™ proprietary commercial software from Advanced Cell Diagnostics, USA and scatter plot representations of PD-L1 protein expression as detected by IHC using H, a Sinobiological antibody recognizing PD-L1 scored by Aperio pixel counting with Imagescope software and I, the 405.9A11 antibody recognizing PD-L1 scored by Aperio pixel counting with Imagescope software.

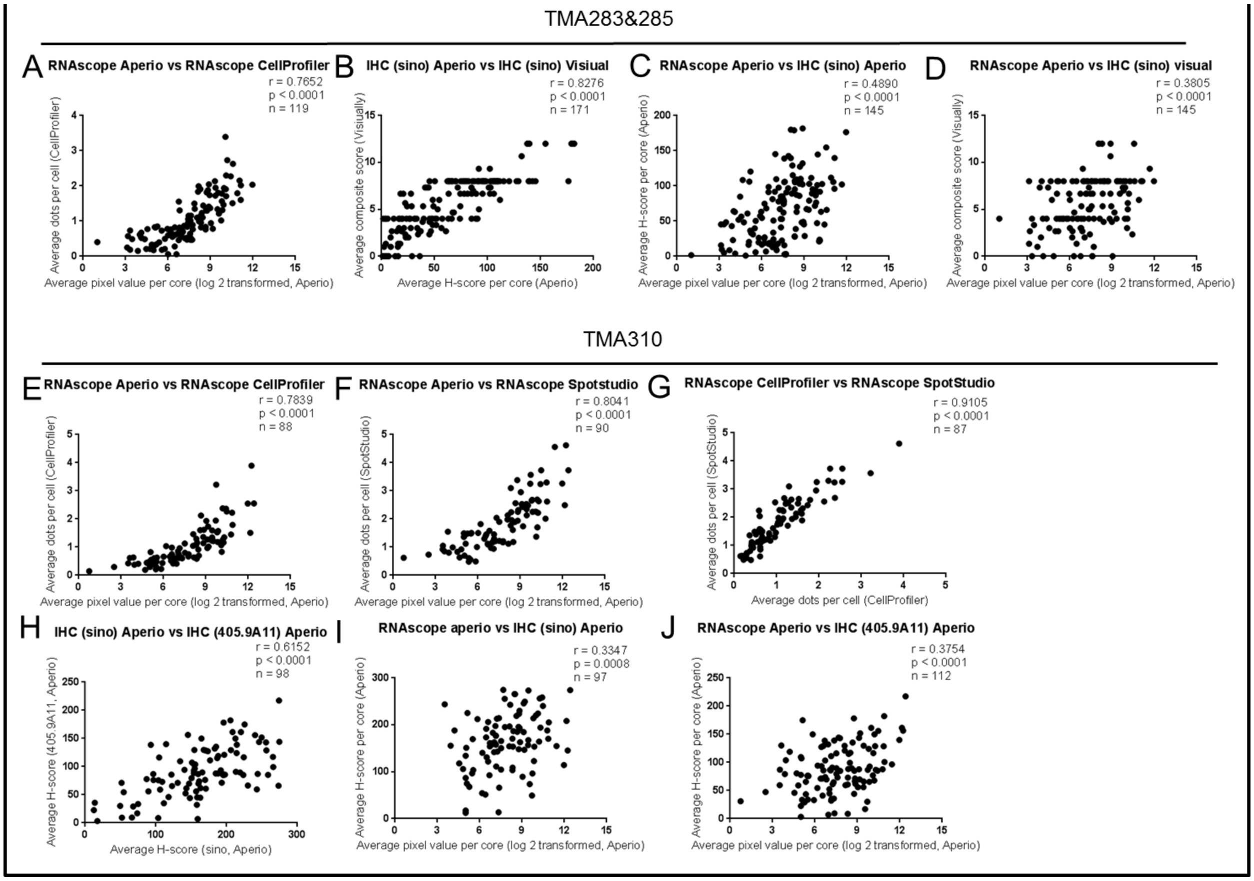

Figure S6: Correlation analysis of PD-L1 RNAscope scored by Aperio, CellProfiler, and Spotstudio™ , as well as PD-L1 protein expression by IHC as scored by Aperio pixel counting with Imagescope software.

Table S1: Clinicopathological characteristics of cases on TMA283 & TMA285 and on TMA310.

Table S2: Immune cell infiltrate in 173 cases of meningioma in TMA283 & 285 by visual light microscopy review (separated into low and high expressors - relative to the median score for each marker for the cases on TMA 283 & 285).

Table S3: Average number of infiltrating immune cells in 291 cases of meningioma.

Table S4: The median value per marker for cases in various cohorts; used for distinguishing low and high expressors.

Table S5: PD-L1 RT-PCR data and PD-L1 RNAscope data scored by Aperio, Spotstudio™ and CellProfiler in 16 cases of meningioma.

Table S6. Clinicopathological characteristics of 6 meningioma primary cell lines.
